# Genome and Transcriptome Analysis of *Ascochyta pisi* Provides Insights into the Pathogenesis of Ascochyta Blight of Pea

**DOI:** 10.1128/spectrum.04488-22

**Published:** 2023-01-16

**Authors:** Na Liu, Chao Liu, Yajing Song, Xingmin Han, Guwen Zhang, Zhijuan Feng, Bin Wang, Yuanpeng Bu, Jinwen Ou, Yaming Gong

**Affiliations:** a State Key Laboratory for Managing Biotic and Chemical Threats to the Quality and Safety of Agro-Products, Key Laboratory of Vegetable Legumes Germplasm Enhancement and Molecular Breeding in Southern China of the Ministry of Agriculture and Rural Affairs, Institute of Vegetables, Zhejiang Academy of Agricultural Sciences, Hangzhou, China; b State Key Laboratory of Rice Biology, Key Laboratory of Molecular Biology of Crop Pathogens and Insects, Department of Plant Protection, Zhejiang University, Hangzhou, China; Institute of Biotechnology

**Keywords:** *Ascochyta pisi*, pea, genome sequencing, comparative genomics, transcriptome, pathogenesis

## Abstract

Ascochyta blight caused by Ascochyta pisi is a major constraint to pea (Pisum sativum L.) production worldwide. Deciphering the pathogenic mechanism of *A. pisi* on peas will help in breeding resistant pea varieties and developing effective approaches for disease management. However, little is known about the genomic features and pathogenic factors of A. pisi. In this study, we first report that *A. pisi* is one of the causal agents of ascochyta blight disease of pea in China. The genome of the representative isolate *A. pisi* HNA23 was sequenced using PacBio and Illumina sequencing technologies. The HNA23 genome assembly is almost 41.5 Mb in size and harbors 10,796 putative protein-encoding genes. We predicted 555 carbohydrate-active enzymes (CAZymes), 1,008 secreted proteins, 74 small secreted cysteine-rich proteins (SSCPs), and 26 secondary metabolite biosynthetic gene clusters (SMGCs). A comparison of *A. pisi* genome features with the features of 6 other available genomes of *Ascochyta* species showed that CAZymes, the secretome, and SMGCs of this genus are considerably conserved. Importantly, the transcriptomes of HNA23 during infection of peas at three stages were further analyzed. We found that 245 CAZymes and 29 SSCPs were upregulated at all three tested infection stages. SMGCs were also trigged, but most of them were induced at only one stage of infection. Together, our results provide important genomic information on *Ascochyta* spp. and offer insights into the pathogenesis of *A. pisi*.

**IMPORTANCE** Ascochyta blight is a major disease of legumes worldwide. *Ascochyta pisi* and other *Ascochyta* species have been identified as pathogens of ascochyta blight. Here, we first report that *A. pisi* causes ascochyta blight of pea in China, and we report the high-quality, fully annotated genome of *A. pisi*. Comparative genome analysis was performed to elucidate the differences and similarities among 7 *Ascochyta* species. We predict abundant CAZymes (569 per species), secreted proteins (851 per species), and prolific secondary metabolite gene clusters (29 per species) in these species. We identified a set of genes that may be responsible for fungal virulence based on transcriptomes *in planta*, including CAZymes, SSCPs, and secondary metabolites. The findings from the comparative genome analysis highlight the genetic diversity and help in understanding the evolutionary relationship of *Ascochyta* species. *In planta* transcriptome analysis provides reliable information for further investigation of the mechanism of the interaction between *Ascochyta* spp. and legumes.

## INTRODUCTION

Peas (Pisum sativum L.) are an annual pulse crop grown worldwide and provide a rich source of protein, minerals, and vitamins for humans and animals. Dry peas are the third most important food legume in the world after dry beans (Phaseolus vulgaris L.) and chickpeas (Cicer arietinum L.), with an average annual production of around 12.9 million metric tons (15.7% of the total production of pulses) during the last decade (1). Meanwhile, the production of green peas is rapidly increasing, reaching around 11.3 million metric tons in 2020, where the majority of the production occurs in China and India, accounting for 57% and 29% of the total production in the world, respectively (1). However, pea production and quality are substantially impacted by biotic and abiotic stresses. Diseases caused by various microorganisms, including fungi, bacteria, and viruses, are some of the main biotic factors limiting the production and quality of peas.

Ascochyta blight (syn., black spot) is the most devastating disease of peas worldwide. Ascochyta blight reduces production, with yield losses of 28 to 88% depending on the environmental conditions and the quality of the peas (2–4). At least seven fungal species have been identified as pathogens of ascochyta blight of pea, including Ascochyta pisi, Ascochyta koolunga, Ascochyta pinodes, Ascochyta pinodella, *Phoma herbarum*, *Phoma glomerata*, and *Boeremia exigua* var. *exigua* (2, 3, 5–7). These pathogens cause similar symptoms on pea plants, with foot rot, black stem and leaf, and pod spot (3). The identification of pathogens was based largely on morphological characteristics such as growth, colony formation, and pigment on media; the shape and size of conidia; conidial characteristics; survival structures; and toxin production during the early days (8, 9). Sequence-based molecular analyses are now routinely used to identify the *Ascochyta* complex and analyze the population structures using marker genes such as the internal transcribed spacer (ITS) region, the RNA polymerase II second-largest subunit (RPB2), the partial β-tubulin (TUB2) gene region, partial 28S large-subunit (LSU) nuclear ribosomal DNA (nrDNA) sequences, and microsatellite markers (5, 7, 9–13). However, marker genes are not able to accurately delimit closely related species in some cases. For example, A. pinodes and A. pinodella are very closely related species that are indistinguishable when using ITS sequence data (14, 15). Whole-genome sequencing will significantly improve our understanding of the taxonomy, genetic diversity, and pathogenic mechanisms of these *Ascochyta* complex members.

Plant-pathogenic fungi commonly form infection structures to penetrate the host plant cell wall during the early stage of the infection process. For example, *A. pinodes* forms infection cushions to penetrate the leaves of peas and Medicago truncatula (3, 16). Various carbohydrate-active enzymes (CAZymes) secreted by fungi to degrade plant cell walls are critical for penetration and virulence. It has been reported that Ascochyta rabiei, the pathogen of ascochyta blight of chickpea, produces cutinase, xylanase, and pectinase to induce chickpea cell death (17–20). These CAZymes are involved in the formation of A. pisi lesions and spreading lesions caused by *A. pinodes* (21). Meanwhile, phytopathogenic fungi produce diverse secondary metabolites (SMs), including polyketides, nonribosomal peptides, terpenes, and alkaloids. Some SMs play essential roles in virulence. For instance, deoxynivalenol, a type B trichothecene, is a critical virulence factor for the pathogenicity of Fusarium graminearum, the causal agent of fusarium head blight (22). A diverse array of phytotoxic metabolites are produced by legume-associated *Ascochyta* and related genera of the Dothideomycetes, and some of them have been claimed to be virulence or pathogenicity factors, such as ascochitine, solanapyrones (A, B, and C), cytochalasin D, and a proteinaceous phytotoxin (20, 23–26). However, few CAZymes and phytotoxins have been investigated for their roles in virulence. The transcriptional patterns of the synthetic genes for CAZymes and SMs during the infection process in plant-pathogenic fungi are largely unknown.

In this study, we sequenced the genome of *A. pisi* HNA23, which was isolated from an infected pea leaf, using PacBio and Illumina high-throughput sequencing technologies. Whole-genome and comparative genome analyses revealed the genomic characteristics of the ascochyta blight complex. We predicted putative CAZymes, secreted proteins, small secreted cysteine-rich proteins (SSCPs), and SM biosynthetic gene clusters (SMGCs) in *A. pisi*, and their transcriptome profiles are described as well. These results provide valuable insight into the taxonomy of fungal pathogens associated with ascochyta blight of pea and the pathogenesis of *A. pisi* on peas.

## RESULTS

### Pathogen isolation and identification.

**FIG 1:**
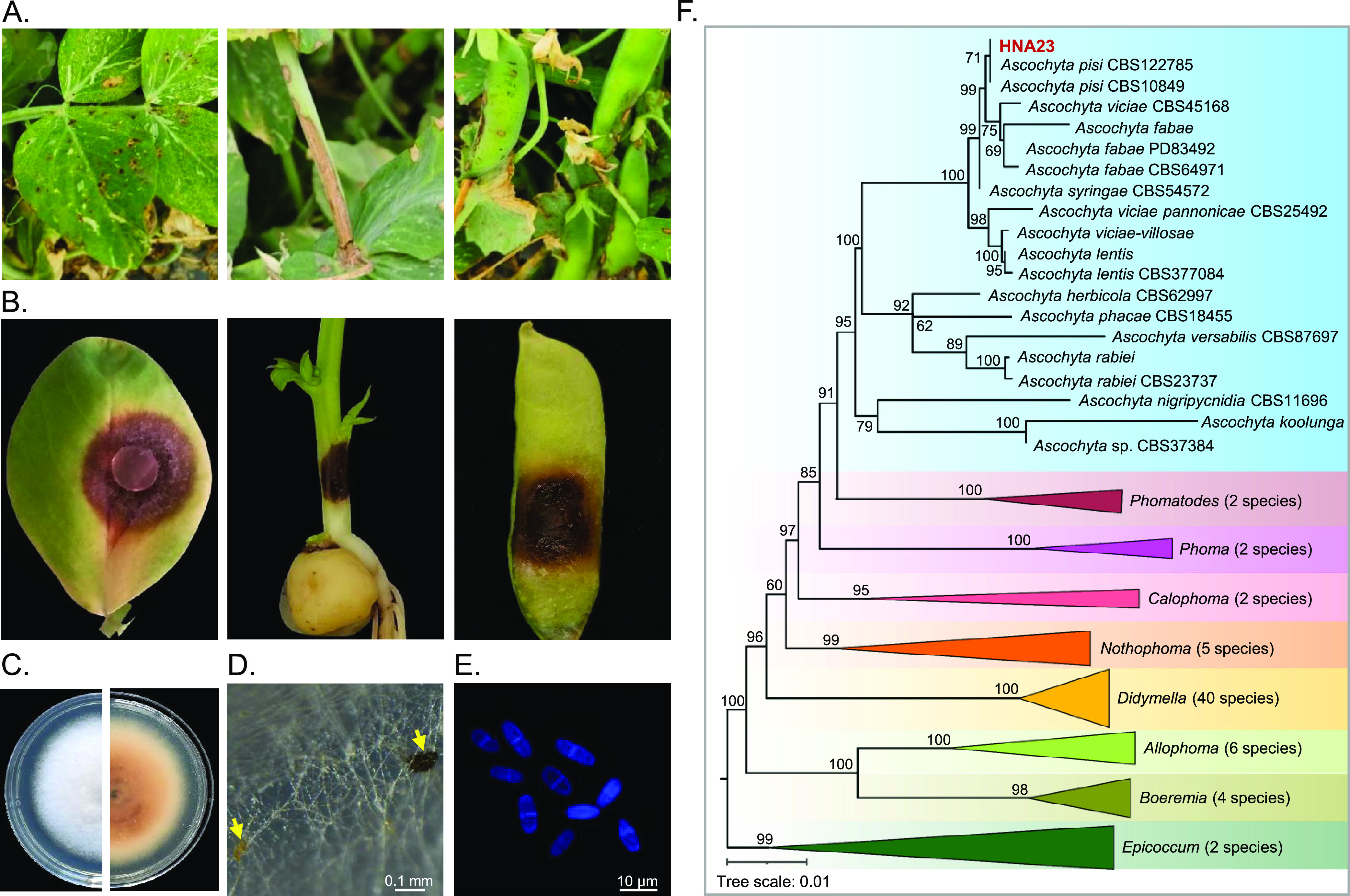
Symptoms of ascochyta blight of pea and identification of the pathogenic strain HNA23. (A) Leaf, stem, and pod lesions of ascochyta blight of pea collected in the field. (B) Symptoms caused by the representative strain HNA23. (C) Colony of HNA23 on complete medium (CM). (D) Pycnidium formation of HNA23 on CM. Typical pycnidia are indicated by yellow arrows. (E) Conidial morphology of HNA23. Conidia were stained by calcofluor white. (F) Phylogenetic tree constructed using the maximum likelihood method based on four widely used housekeeping genes as markers (ITS, RPB2, TUB2, and LSU).

Infected pea leaves, stems, and pods with typical symptoms of ascochyta blight were collected from the field (30.25N, 120.21E) (Fig. 1A). A total of 50 fungi were isolated; subsequently, an analysis of their pathogenicity was conducted based on Koch’s postulates (Fig. 1B). A dominant fungal colony, HNA23, was selected as the representative isolate for further study. HNA23 formed dense white and felty colonies on a complete medium (CM) agar plate, which became gray to dark (Fig. 1C). It produced yellow or dark pycnidia under induction conditions (Fig. 1D). Conidia are greatly variable in shape and size, being fusiform or ellipsoidal, mainly uniseptate (~80%) but sometimes aseptate (~20%) (*n* = 120), and 9.0 to 12.3 by 3.4 to 5.1 μm (Fig. 1E). A multilocus phylogenic tree was constructed to determine the phylogenetic placement of HNA23 based on the DNA sequences of four loci (ITS, RPB2, TUB2, and LSU). HNA23 was identified as *A. pisi* according to the maximum likelihood (ML) tree (Fig. 1F; see also Fig. S1 in the supplemental material).

### High-quality draft genome assembly and annotation of HNA23.

**FIG 2:**
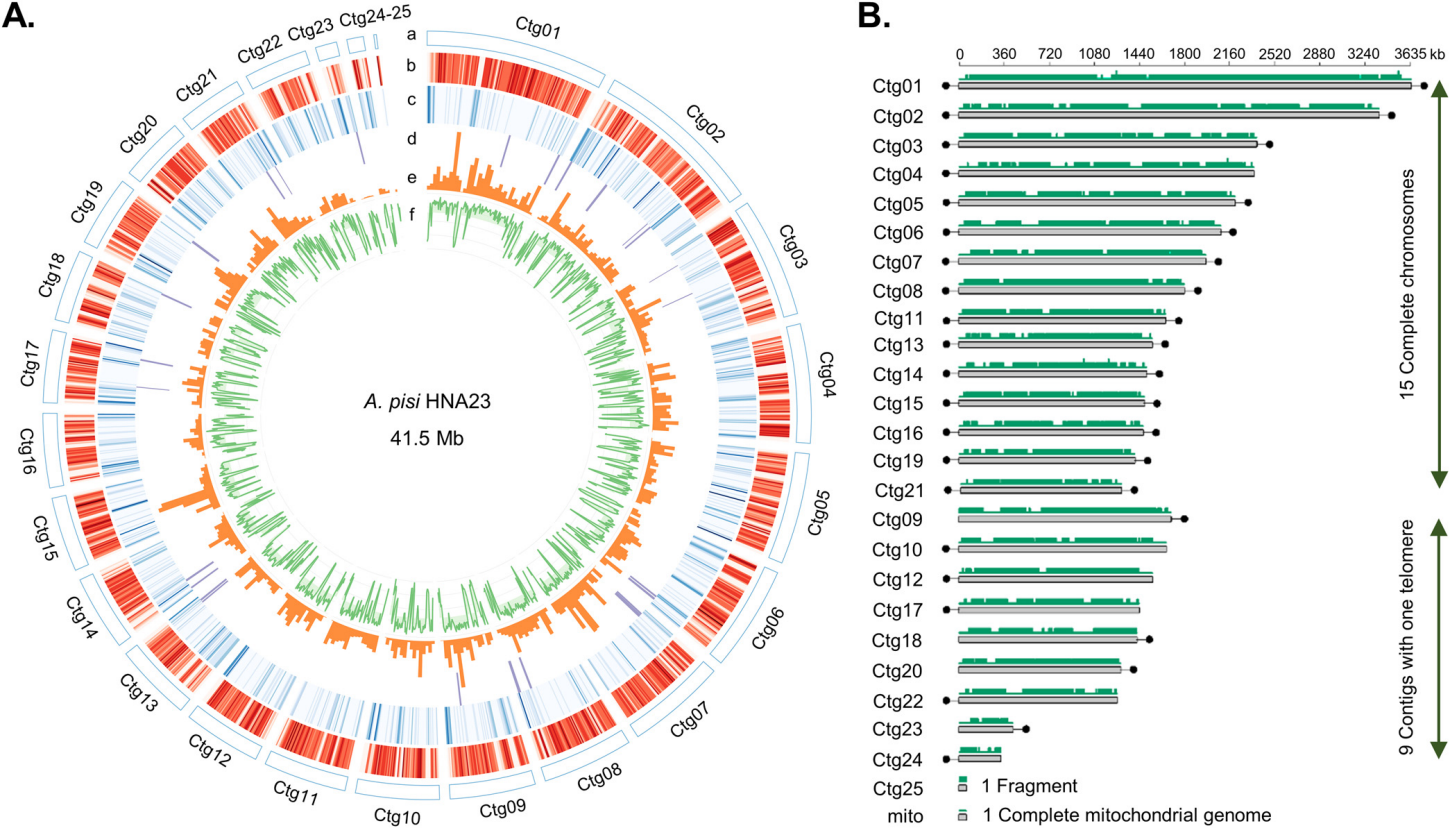
Overview of the *A. pisi* HNA23 genome. (A) Circos plot of key genome features of *A. pisi* HNA23. The circles labeled from the outside are as follows: 25 contigs with the size scale (a), gene density (red) (b), repetitive DNA density (blue) (c), location of the predicted biosynthetic gene clusters for secondary metabolites (purple) (d), coverage of RNA-seq reads on the genome (orange) (e), and GC content (percent) (green) (f). All statistics are based on 25-kb nonoverlapping windows. (B) Genome contigs of *A. pisi* HNA23 assembled by the combination of PacBio sequencing and correction with Illumina short reads. Nuclear contigs are labeled as Ctg01 to Ctg25, and the complete mitochondrial genome is labeled as mito. Gene-dense regions of the genome are shown as green-shaded blocks, joined by gene-sparse. Telomeres are indicated in the contigs by black dots.

The genome of HNA23 was assembled using a combination of PacBio sequencing and correction with Illumina short reads. A total of 657,056 high-quality clean reads (~9.11 Gb, with an estimated 200-fold coverage depth) with an average length of 13.8 kb were obtained from the PacBio data after quality control (Table 1). Meanwhile, approximately 5.95 Gb of Illumina short reads were also generated. The assembled HNA23 genome consisted of 25 contigs with an *N*_50_ of 1.7 Mb and a high-quality assembly of 41,458,304 bp (Fig. 2A). As shown in Fig. 2B, we found 15 complete nuclear chromosomal contigs with telomere sequences at both ends (contigs 1 to 8, 11, 13 to 16, 19, and 21) and 9 partial chromosomal contigs with 1 telomere. In addition, a large contig without a telomere (contig 25) and a single mitochondrial genome of 53,248 bp were assembled. Using the dothideomycetes_odb10 data set of the Benchmarking Universal Single-Copy Orthologs (BUSCO) database as a measure of genome completeness, we found that 99.5% of the expected single-copy orthologs (3,786) were found as single copies in the HNA23 genome (Table 1), indicating that the genome assembly of HNA23 was complete.

**TABLE 1:** Features of the *A. pisi* HNA23 assembled genome

Feature	Value for HNA23
Genome size (bp)	41,458,304
No. of sequence reads	657,056
Total sequenced bases (Gb)	9.1
Coverage depth (fold)	220
No. of contigs	25
Largest contig size (bp)	3,633,783
Contig *N*_50_ (bp)	1,699,839
Contig *L*_50_	9
GC content (%)	48.13
No. of putative genes	11,001
No. of protein-encoding genes	10,796
Total length of protein-encoding genes (bp)	17,750,684
Gene density (no. of genes/Mb)	265
No. of complete BUSCOs	3,768
No. of fragmented BUSCOs	2
No. of missing BUSCOs	16
No. of rRNAs	48
No. of tRNAs	157
Repeat rate (%)	24.84
Mitochondrial genome size (bp)	53,248
No. of predicted secondary metabolite clusters	26
No. of predicted CAZymes	555
No. of predicted secreted proteins	1,008

The average GC content and repeat rate were 48.13% and 24.84% in the HNA23 genome, respectively. A total of 11,001 putative genes with an average gene length of 1,644 bp were identified. The total length of these predicted genes was 17,750,684 bp, accounting for 42.82% of the genome. The biological functions of these predicted proteins were further analyzed based on the Gene Ontology (GO), Kyoto Encyclopedia of Genes and Genomes (KEGG), and Eukaryotic Orthologous Groups (KOG) databases. GO analysis indicated that 8,011 (74.20%), 7,325 (67.85%), and 7,569 (70.10%) of the predicted proteins were assigned to cellular components, molecular function, and biological processes, respectively (Fig. S2). KEGG results showed that 2,781 proteins were enriched in signal transduction, transport and catabolism, and 225 other pathways belonging to 4 categories (Fig. S3). Moreover, KOG analysis revealed that the “carbohydrate transport and metabolism” category (G) (9.6%) had the highest number of proteins, followed by the “posttranslational modification, protein turnover, chaperones” category (O) (8.2%) and the “amino acid transport and metabolism” category (E) (6.7%), excluding the unknown function category (Fig. S4). In addition, 157 tRNAs and 48 rRNAs were predicted in the genome assembly (Table 1).

### Phylogenomic statistics and ortholog analysis of *Ascochyta* species.

**FIG 3:**
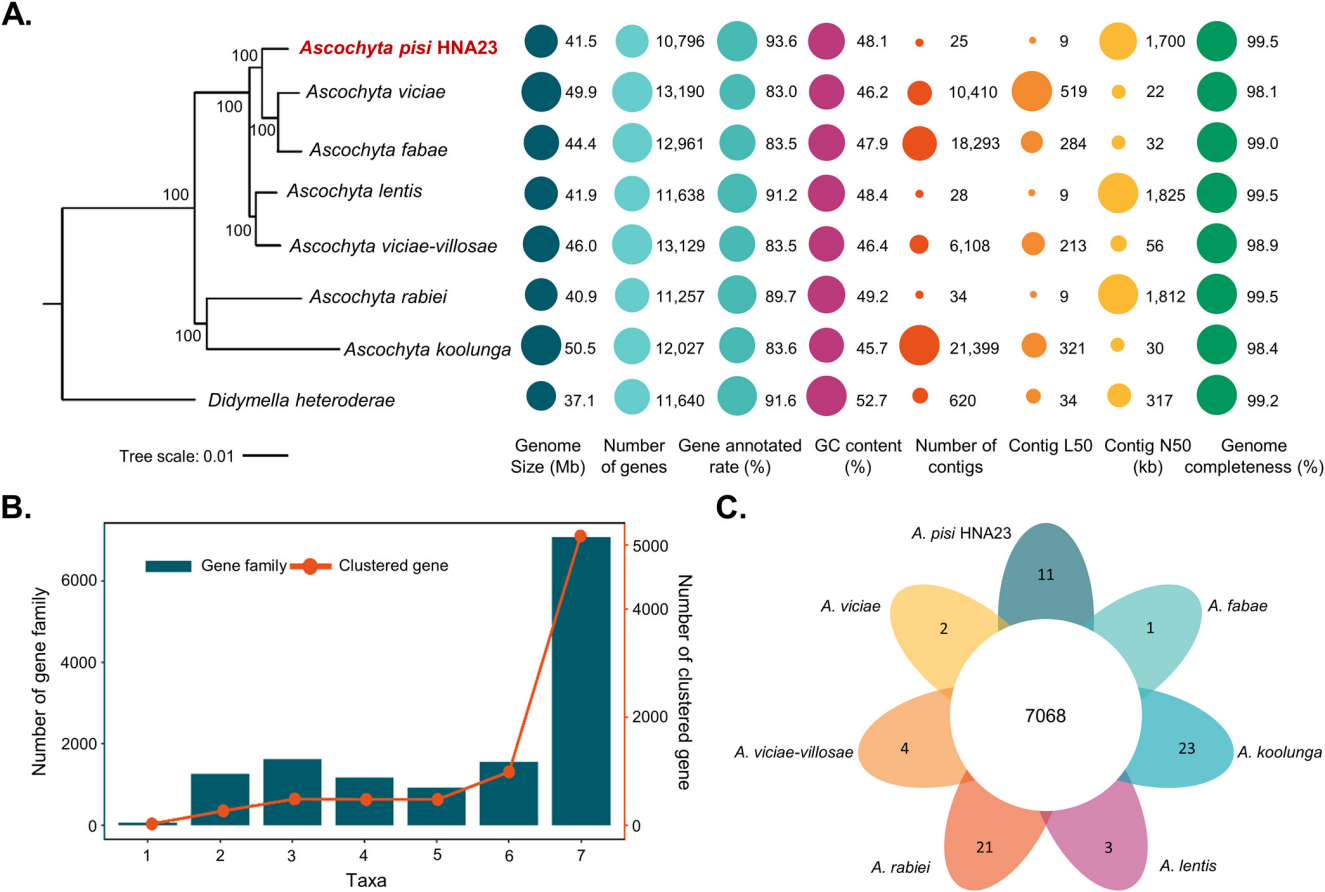
Phylogeny and genome comparison analysis between HNA23 and six other *Ascochyta* species. (A) Phylogenetic tree constructed using a concatenation single-model (LG+G4) approach based on maximum likelihood analysis of 3,783 single-copy genes. The genome of *Didymella heteroderae* was used as the outgroup. Eight bubble plots show key genome features and sequencing quality parameters. The bubble sizes have been relatively scaled to each panel and are not comparable across panels. The genome of *Didymella heteroderae* was used as the outgroup. (B) Numbers of gene families and genes clustered in the corresponding families. The number of taxa is shown below the *x* axis. (C) Flower-shaped Venn diagram of protein conservation in different species. The numbers of all conserved families in the seven *Ascochyta* species and unique families preserved in each species are indicated at the center and tips, respectively.

To provide an overview of the genomic features and the relationships among the sequenced species of *Ascochyta*, we compared the HNA23 genome to 6 other available sequenced *Ascochyta* species genomes. The qualities of the genome assemblies were compared based on genome size, GC content, and the number of predicted proteins (Fig. 3A). Although the draft genomes of *Ascochyta viciae-villosae*, *A. viciae*, *A. fabae*, and *A. koolunga* were sequenced with Illumina short reads, the genomes covered 98.9%, 98.1%, 99.0%, and 98.4% of the BUSCOs, respectively. We thus concluded that the individual genomic annotations of the above-mentioned species were of a high enough quality for comparisons of the gene contents despite the large number of scaffolds. The genome sizes of *Ascochyta* species varied from 40.91 to 50.53 Mb (average of 45.03 Mb). The genome of *A. pisi* is the second smallest genome of the members of the genus. We next constructed a phylogenetic tree based on 3,783 single-copy orthologs of *A. pisi* HNA23 and 6 other *Ascochyta* species, with Didymella heteroderae as the outgroup (Fig. 3A). *A. pisi* HNA23 was a sister group to *A. viciae* and *A. fabae*, with 100% bootstrap support, which was consistent with the results of the phylogenetic analyses based on the DNA sequences of four loci (Fig. 1F).

We next analyzed the core genome shared by *Ascochyta* species and species-specific genes. A total of 13,621 families (GF00001 to GF13621) containing 81,545 genes were identified and annotated through gene family structure analysis. The results showed that 7,068 essential families were shared by 7 *Ascochyta* species and 65 species-specific families (Fig. 3B and C; Table S1). Notably, 201 unique genes in HNA23 were analyzed using multiple functional databases. Functional analysis showed that 30 annotated unique genes were enriched in cellular components and molecular functions. The functions of the other 171 unique genes were unknown.

### Carbohydrate-active enzymes are abundant in *Ascochyta* spp. and are closely related to infection.

**FIG 4:**
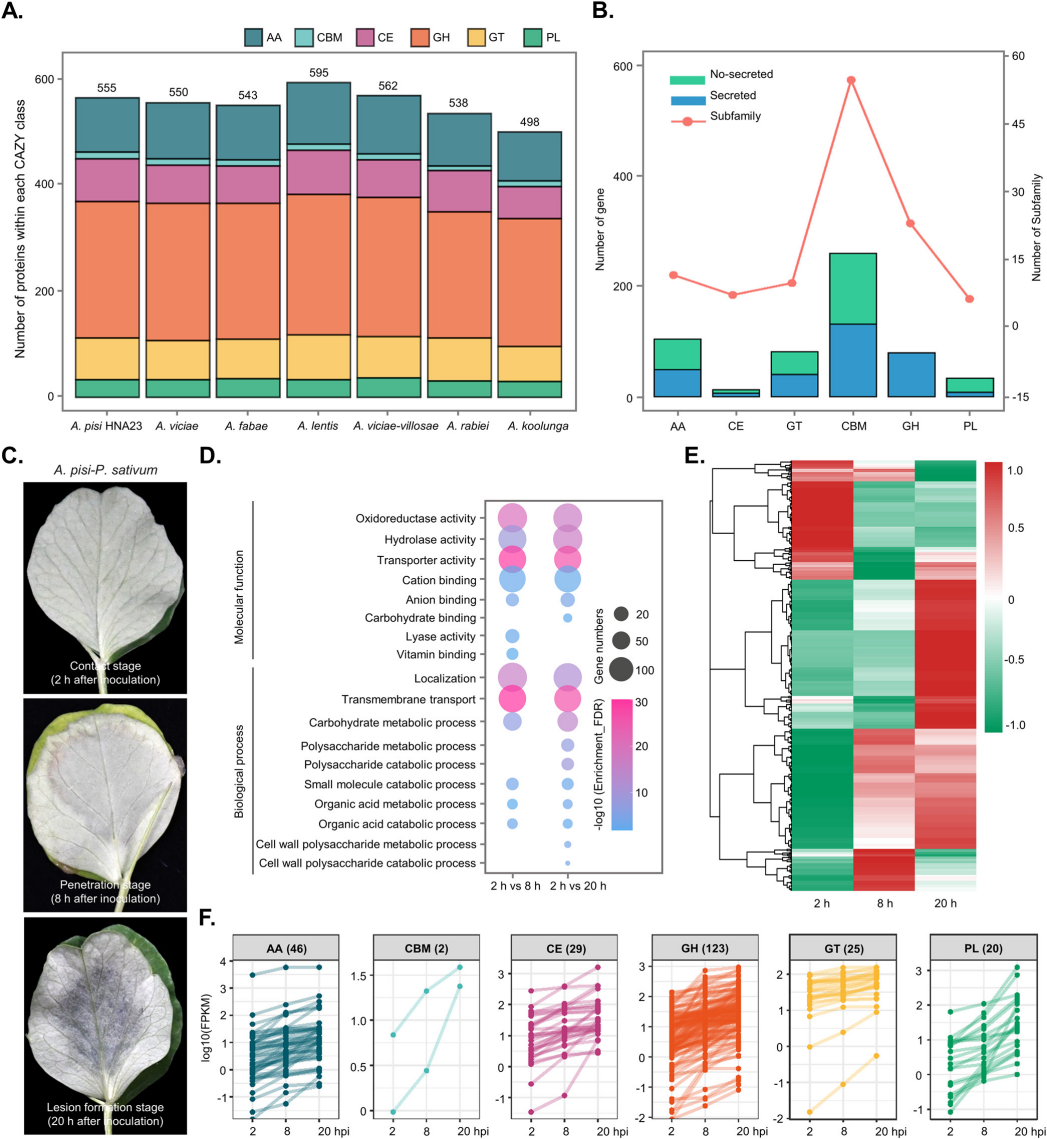
Carbohydrate-active enzymes (CAZymes) in *A. pisi* HNA23. (A) Total number of CAZymes in each *Ascochyta* species distributed into six categories of enzyme activity: auxiliary activities (AA), carbohydrate-binding molecules (CBM), carbohydrate esterases (CE), glycoside hydrolases (GH), glycosyltransferases (GT), and polysaccharide lyases (PL). (B) Number of CAZymes and their relevant families. (C) Disease development of *P. sativum* infected by *A. pisi*. Representative infected pea leaves at three sampling time points are shown. (D) Gene Ontology enrichment analysis of the upregulated genes of *A. pisi* at the penetration and lesion formation stages, in comparison to the contact stage. FDR, false discovery rate. (E) Heat map of the normalized FPKM (fragments per kilobase of transcript per million fragments mapped) values of differentially expressed genes encoding CAZyme proteins at different infection stages. The Z-score represents the deviation from the mean by standard deviation units. Red and green indicate upregulated and downregulated genes, respectively. (F) Expression of genes encoding CAZymes with constantly increasing patterns during the infection process.

As plant-pathogenic fungi, *Ascochyta* spp. require various carbohydrate-active enzymes (CAZymes) to degrade the components of plant cell wall polysaccharides such as cellulose, hemicellulose, and pectin. A total of 3,841 CAZymes were predicted for the 7 *Ascochyta* species (average of 569/species) using the CAZy database (Fig. 4A; Table S1). This is quite rich compared with other plant-pathogenic fungi (27). *A. pisi* HNA23 harbored 555 CAZymes, which is a relatively large number in comparison to those in other *Ascochyta* spp., excluding *A. lentis* and *A. viciae-villosae* (Fig. 4A). The CAZymes in *A. pisi* HNA23 were clustered into six classes with different family distributions and numbers of genes (Fig. 4B). Glycoside hydrolases (GHs) (258 genes) were the largest class, followed by auxiliary activities (AAs) (106), carbohydrate esterases (CEs) (75), polysaccharide lyases (PLs) (34), glycosyl transferases (GTs) (77), and carbohydrate-binding modules (CBMs) (5) (Table S2).

To investigate the potential contribution of CAZymes in HNA23 to interactions with its host, we performed transcriptome sequencing (RNA-seq) analysis of the transcriptional profiles of HNA23 during interactions with pea leaves. Infected samples from three infection stages were collected using the sandwiched sample preparation method, including 2 h (contact stage [CS]), 8 h (penetration stage [PS]), and 20 h (lesion formation stage [LFS]) postinoculation (Fig. 4C). After Illumina sequencing, the fragments per kilobase per million mapped read (FPKM) values for *A. pisi* transcripts were calculated using the reads mapped onto the HNA23 genome and were compared between replicates to determine biological deviations. Approximately, 10.4 million to 17.7 million clean reads were generated for each sample, and 9.0 million to 14.4 million of these reads were mapped to genes in the HNA23 genome for further analysis (Table S3). A total of 10,486 genes (97.1% of the genes in HNA23) were detected in all samples. Pearson’s correlation coefficient (*R*) values were highly significant and ranged from 0.84 to 0.99 (*P < *0.001) among the replicates. Based on the FPKM values, a heat map analysis was performed to visualize the profiles of differentially expressed genes (DEGs) during infection processes. As shown in Fig. S5A, the expression levels of a large number of genes displayed dramatic changes during the interaction between HNA23 and peas.

Further, an absolute log_2_ fold change value of ≥1 was set as a cutoff standard to identify DEGs between different infection stages, including the CS (2 h postinoculation [hpi]) versus the PS (8 hpi) and the CS (2 hpi) versus the LFS (20 hpi). Totals of 2,643 (1,391 upregulated and 1,252 downregulated) and 2,925 (1,575 upregulated and 1,350 downregulated) DEGs were identified in the comparison of the CS (2 hpi) versus the PS (8 hpi) and the CS (2 hpi) versus the LFS (20 hpi), respectively (Fig. S5B and C and Table S4). Among them, the mRNA expression levels of 1,022 genes, including 19 unique genes, were significantly elevated during both the PS and LFS in comparison to those during the CS (Fig. S5D, Fig. S6, and Table S5).

Next, the upregulated DEGs in the two comparison groups were classified into GO categories. The results indicated that these genes were markedly enriched in oxidoreductase activity, hydrolase activity, transport activity, and other terms. Notably, genes involved in oxidoreductase activity, hydrolase activity, and carbohydrate metabolic processes were greatly induced in both the PS and LFS. In addition, genes participating in polysaccharide catabolic and metabolic processes were specifically upregulated in the LFS (Fig. 4D). Genes encoding CAZymes showed dramatic dynamic changes. Many CAZymes were induced during the interaction, especially in the PS and LFS (Fig. 4E). Importantly, we found that the expression of 245 genes encoding CAZymes (~44% of the total CAZymes) constantly increased with the development of disease (Fig. 4F; Table S6). Together, these results implied that the proteins responsible for penetrating and digesting plant cell walls played important roles during the *A. pisi*-P. sativum interaction, especially CAZymes.

### The secretome expressed *in planta* and small secreted cysteine-rich proteins in *A. pisi*.

**FIG 5:**
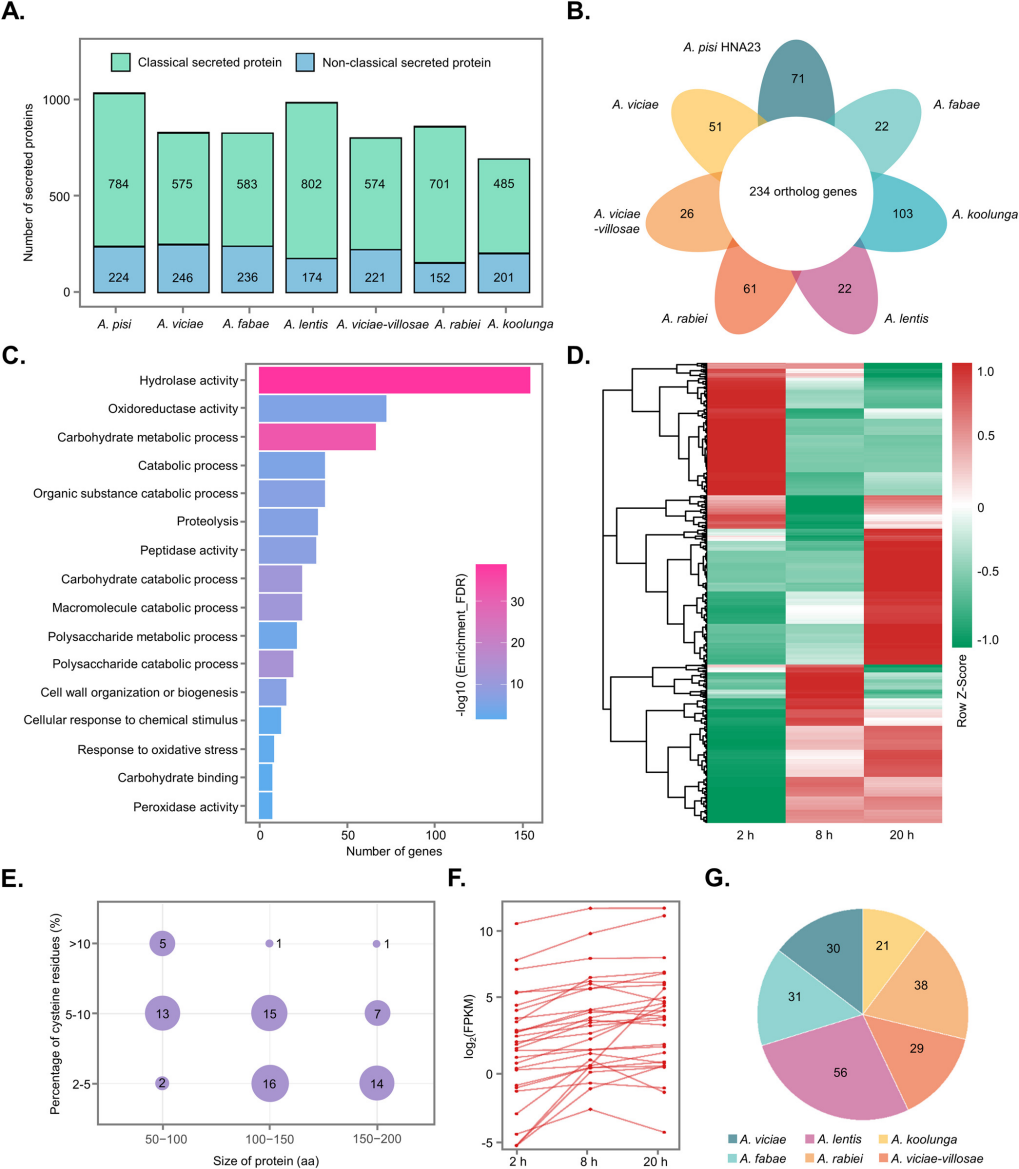
Prediction and analysis of the *A. pisi* secretome. (A) Total number of secreted proteins in each *Ascochyta* species. (B) Flower-shaped Venn diagram showing the conservation of secreted proteins in different species. The numbers of all conserved orthologs in 7 *Ascochyta* species and unique secreted proteins in each species are indicated at the center and tips, respectively. (C) Gene Ontology enrichment analysis of the *A. pisi* secretome. (D) Heat map of the normalized FPKM values of differentially expressed genes encoding secreted proteins at different infection stages. The Z-score represents the deviation from the mean by standard deviation units. Red and green indicate upregulated and downregulated genes, respectively. (E) Distribution of protein sizes and cysteine percentages of small secreted cysteine-rich proteins (SSCPs) identified in *A. pisi* HNA23. (F) Expression patterns of upregulated SSCPs during the infection process. (G) *A. pisi* SSCP orthologs in other *Ascochyta* species. The number of orthologs in each species is indicated.

Plant pathogens secrete abundant proteins during their interactions with hosts, which are critical for successful infection by pathogens through various mechanisms such as the suppression of host immunity by effectors (28). Therefore, putative secreted proteins and their transcriptional patterns were analyzed in *A. pisi*. A combination of three approaches based on SignalP v5.0, TMHMM v2.0, and SecretomeP 2.0 was used to predict the secretome of *Ascochyta* spp. As shown in Fig. 5A, an average of 851 putative secreted proteins were identified in this species, in which 75% of the proteins were classical secreted proteins with signal peptides (Table S7). Analysis of the unique and shared orthologous proteins revealed that 234 orthologs were highly conserved in all 7 species, and 356 proteins were species specific (Fig. 5B). The *A. pisi* HNA23 genome encodes 1,008 putative secreted proteins (9.5% of the total predicted proteins in the genome), including 789 classical and 237 nonclassical secreted proteins. Among them, 256 secreted proteins are CAZymes. GO analysis indicated that these genes were enriched mainly in three GO categories: hydrolase activity, oxidoreductase activity, and carbohydrate metabolic processes (Fig. 5C). This was consistent with the GO enrichments of upregulated genes in the HNA23 genome during infection (Fig. 4D). Importantly, most genes (98%; 988 out of 1,008) encoding the above-mentioned putative secreted proteins were expressed during the infection process in at least one tested stage (Fig. 5D; Table S8).

Pathogenic fungi produce diverse small secreted cysteine-rich proteins (SSCPs) as effectors to manipulate host plant immunity for successful infection (29). However, this group of proteins has not been analyzed in *Ascochyta* spp. Therefore, we further identified SSCPs and analyzed their transcriptional profiles during the interaction between *A. pisi* and peas. Based on the criteria that a secreted protein consists of ≤200 amino acids (aa) and contains ≥2% cysteine residues, a total of 74 SSCP candidates were identified in HNA23, accounting for 7.3% of the total secreted proteins (Table S9). These 74 SSCPs ranged in size from 67 to 197 aa, with the majority (54) being >100 aa, and the percentage of cysteines was 2 to 12%, with most (42) containing >5% (Fig. 5E). Most of them (55 genes) were identified as a hypothetical protein or an uncharacterized protein. BLAST conserved domain searches suggest that 14 SSCPs have homology to functional domains in the Pfam database, including a LysM domain (Apg4484 protein) (Table S9). RNA-seq data showed that 61 (82%) SSCPs were expressed during infection; especially, 29 SSCPs were induced during either the penetration or the lesion formation stage (Fig. 5F; Table S9). Sequence analysis indicated that 18 (24%) SSCPs were *A. pisi* specific because they all lack homologs in the other six *Ascochyta* spp. (Fig. 5G). These *A. pisi*-specific SSCPs showed expressional patterns similar to those of conserved SSCPs in all 7 tested *Ascochyta* species (Fig. S7). Overall, these results suggested that the secretome of *A. pisi* might function in facilitating fungal colonization, the degradation of host plant tissues, resistance to oxidative stress, and suppression of host immunity, especially through SSCPs.

### Secondary metabolite profile of *A. pisi* compared with those of other *Ascochyta* spp.

**FIG 6:**
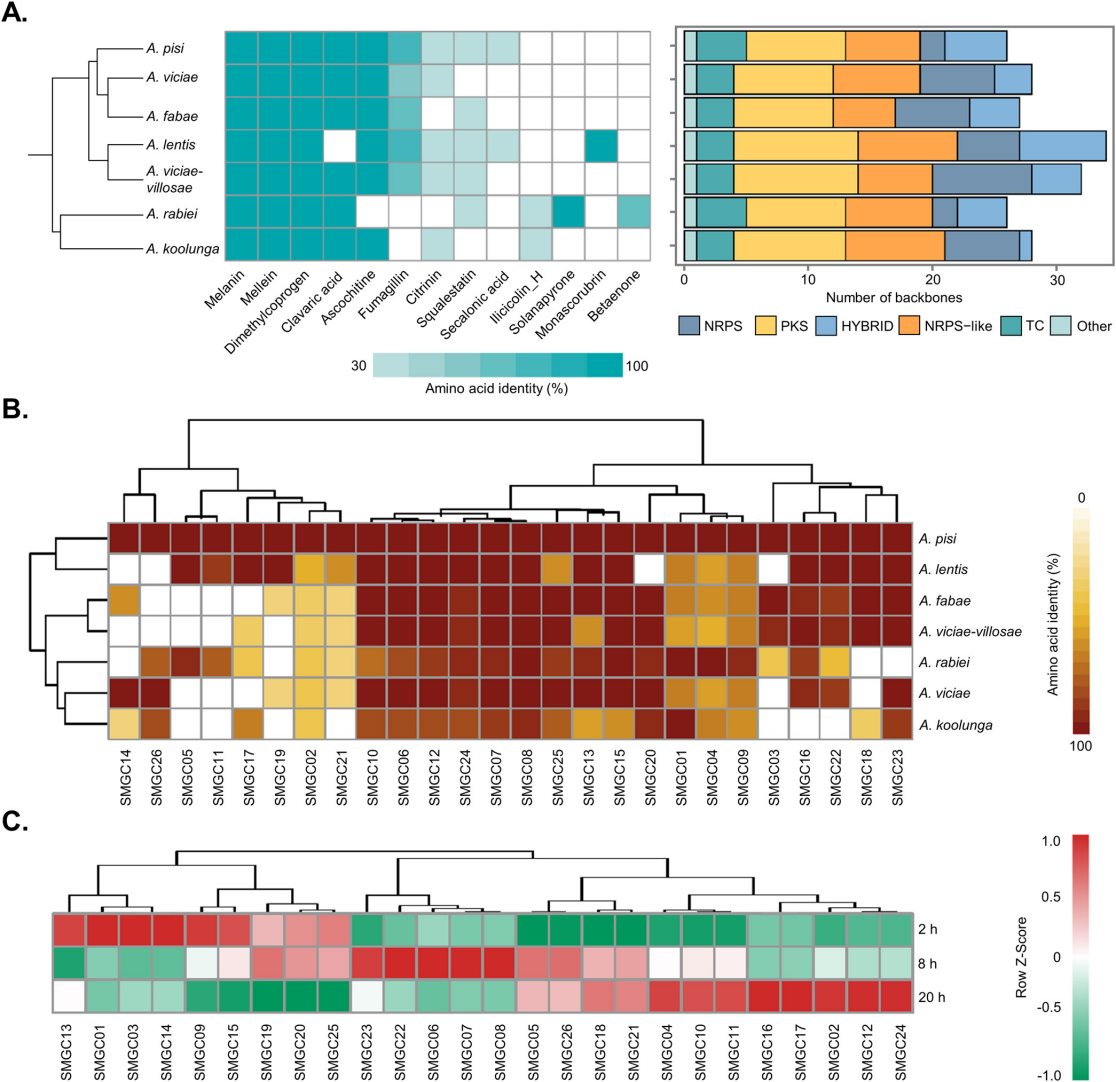
Secondary metabolic gene clusters and their expression profiles during infection. (A) Diversity of secondary metabolic gene clusters (SMGCs) in *Ascochyta* spp. The matrix indicates the presence and absence of SMGC families coupled with known clusters for each species. The predicted secondary metabolite genes for each species are divided by the backbone enzymes. NRPS, nonribosomal peptide synthetase; PKS, polyketide synthase; HYBRID, backbone gene containing domains from the NRPS and PKS backbones; TC, terpene cyclase. (B) Overview of the SMGCs predicted in *A. pisi* and their homologs in other species. (C) Expression profiles of backbone genes in each SMGC at different infection stages. The Z-score represents the deviation from the mean by standard deviation units. Red and green indicate upregulated and downregulated genes, respectively.

Pathogenic fungi produce many secondary metabolites (SMs) to facilitate infection and ensure their fitness. To quantitatively examine the potential for SM production, SM biosynthetic gene clusters (SMGCs) were predicted using fungiSMASH for all 7 *Ascochyta* spp., and a total of 201 SMGCs were identified (~29/species). We next qualitatively assessed potential SM production by analyzing the amino acid similarity with verified characterized clusters in the antiSMASH database. The results indicated that 12 SMGCs showed high similarity (>30% identity) to known compounds. Especially, biosynthetic gene clusters responsible for melanin, mullein, and dimethylcoprogen production were identified in all of these species (Fig. 6A).

*A. pisi* has 26 predicted SMGCs, including 8 PKS (polyketide synthase), 2 NRPS (nonribosomal peptide synthetase), 6 NRPS-like, 5 hybrid (backbone gene containing domains from the NRPS and PKS backbones), and 4 TC (terpene cyclase) SMGCs and 1 biosynthetic gene cluster for indole production (Fig. 6A; Table S10). An overview of the similarity of backbone proteins of the SMGCs between *A. pisi* and the other 6 tested *Ascochyta* species indicated that 13 SMGCs were highly conserved, with an average amino acid identity of 86% for the 7 tested species. *A. pisi* has no species-specific SMGC and showed SMGC patterns similar to those of *A. lentis* (Fig. 6B). We next analyzed the transcriptional profiles of these SMGCs during the interaction between *A. pisi* and peas based on the above-described RNA-seq data. As shown in Fig. 6C, the expression of SMGCs was induced in at least one infection stage, indicating that secondary metabolism was involved in fungal pathogenicity.

## DISCUSSION

Until now, at least seven fungal species have been identified as the causative agents of ascochyta blight of pea (2, 3, 5–7). They are soilborne, pathogenic fungi and survive as chlamydospores, mycelia, or sclerotia in soil, and they can coexist on peas or exist independently from each other (9). However, the component pathogens of this disease complex are varied in different pea-growing regions. For instance, *A. pinodes* and *Phoma koolunga* are the dominant pathogens of the ascochyta blight complex in the Canadian prairies and southern Australia, while *A. pisi* is the predominant causal pathogen in North America, western Canada, and southern France (4, 30, 31). These geographical population structures of the ascochyta blight complex might be determined by the precipitation temperature, the ability for chlamydospore formation, and the exchange of seed germplasms (9, 13, 31). In our previous study, *A. pinodes* was isolated from ascochyta blight lesions on field peas in Zhejiang Province, China (3). Here, we found that *A. pisi* was another causal agent of ascochyta blight of pea. To our knowledge, this is the first report of *A. pisi* as a pathogen of ascochyta blight of field peas in China, suggesting that pathogens of this disease in China could be as complex as that in other countries.

The history of the taxonomy of *Ascochyta* has been unstable due to the lack of clear morphological characteristics of this genus. Previously, *Ascochyta* classification was based only on the ratio of septate conidia on artificial medium or *in planta* (32). However, septum formation by conidia is a continuous developmental process, and the ratio of septate conidia is constantly changing. For example, both uniseptate and aseptate conidia were observed, and the ratio was equivalent in HNA23 on this occasion, which seriously affected the identification of the taxonomy. The application of phylogenetic species criteria greatly improves the accuracy of the identification of *Ascochyta* spp. by using multiple housekeeping genes, such as ITS, RPB2, TUB2, and LSU (3, 10, 33). Combining morphological and phylogenetic criteria, HNA23 was identified as *A. pisi*. In agreement, the phylogenetic tree constructed with 3,783 monocore gene markers in *Ascochyta* spp. further confirmed that the HNA23 strain belongs to *A. pisi*. Therefore, whole-genome sequencing and the phylogenetic tree based on monocore gene markers will significantly improve our understanding of the taxonomy. In addition, we found that several species-specific genes existed in this genus, which are also potential marker genes for taxonomy and diagnostic tools in the future. The teleomorph of *A. pisi* was placed within the genus *Didymella* as *D. pisi* based on the teleomorphic characteristics induced under laboratory conditions and the phylogenetic tree of RPB2 (11). However, we found that 20 tested strains of *Ascochyta* spp. and 40 tested strains of *Didymella* species were significantly placed on different branches, indicating that the genetic distance of these genera was relatively far. Moreover, the monocore gene phylogeny of the available genomes also confirmed this conclusion. Therefore, intensive sampling of the appropriate taxa and type material and additional whole-genome sequencing will be required to accurately classify the teleomorph of *A. pisi*.

The genomic characteristics and mode of pathogenesis of *A. pisi* are unknown in the ascochyta blight complex of pea. In this study, the genome of *A. pisi* and its transcriptome during infection processes were investigated. The whole genome of *A. pisi* HNA23 was sequenced, assembled, and analyzed. The high-quality assembly size was 41.5 Mb, which was within the range of those of the available genomes of *Ascochyta* spp. (40.9 to 50.5 Mb) (34, 35). To penetrate plant cell walls and use host components for their growth, pathogenic fungi secrete diverse CAZymes during infection. A. rabiei secretes cutinase, xylanase, and pectinase to degrade the host barrier (36). In agreement with this, a total of 555 putative CAZymes were predicted in *A. pisi*, and 44% of the total CAZymes were constantly increased with the development of infection, suggesting that CAZymes facilitate fungal infection and host colonization and that the function of CAZymes is highly conserved in pathogenic fungi. In addition to CAZymes, fungi also secrete effectors to manipulate host immunity. It has been reported that the nuclear effector ArPEC25 of *A. rabiei* manipulated the lignin biosynthesis of chickpea for invasion by targeting the transcription factor CaβLIM1a (37). The effector AlAvr1 from *A. lentis* mediated the host cultivar specificity of ascochyta blight of lentil (38). Homolog analysis found that the ArPEC25 homologs were highly conserved, with over 80% amino acid identities in all available genomes of *Ascochyta* spp. except for *Ascochyta koolunga*. However, AlAvr1 is a species-specific effector. Recently, SSCP-derived effectors produced by plant-pathogenic fungi have attracted broad attention and have been demonstrated for a number of ascomycete pathogens (29) but have not been explored in *Ascochyta* spp. In this study, we analyzed a total of 74 SSCPs encoded in the genome of *A. pisi* and found that the expression of 29 SSCPs was induced during fungal infection. It is worth noting that one SSCP (Apg4484) is a putative LysM effector (39), and its expression was increased during the lesion formation stage. Therefore, we have identified 245 CAZymes and 29 SSCPs encoded in the genome of *A. pisi* that may play roles in fungal virulence on peas, through genomic and transcriptional profiling analyses. Further investigation of these CAZymes and potential effector-like proteins may identify new virulence factors of *A. pisi* and improve our understanding of the molecular mechanism of the interaction between *A. pisi* and peas.

To successfully infect a host, fungal pathogens need to counteract the reactive oxygen species (ROS) and fungitoxic compounds produced by host plants as a defense, such as phenylpropanoids, terpenoids, phytoalexins, and glucosinolates (17). Similar to the *A. rabiei*-chickpea interaction (17), upregulated genes in *A. pisi* were largely enriched in the GO functional category “oxidoreductase activity” during infection, which detoxifies ROS to improve fungal survival in the aggressive environment created by the plant. Most pathogens have evolved abilities to overcome the phytoalexins produced by their host plants during the arms race between plant hosts and their associated pathogens (40). Pisatin is the major phytoalexin produced by peas against pathogenic microbial attack. The enzyme for detoxifying pisatin, pisatin demethylase (PDA), has been well studied in Nectria haematococca MPVI. The ΔPDA1 mutant increased the sensitivity to pisatin and reduced virulence on peas (41, 42). Apg7855 of *A. pisi* shares high amino acid identity (43%) with PDA of N. haematococca and exhibits an upregulated pattern during infection, suggesting that Apg7855 may have the ability to detoxify pisatin in peas. Meanwhile, fungal pathogens produce several phytotoxins to kill plant cells to promote infection. Until now, about 20 phytotoxins have been identified in *Ascochyta* spp. (26, 43). For example, ascochitine and its precursor ascosalitoxin produced by *A. pisi* exhibit phytotoxicity and are associated with fungal virulence (44). However, very few biosynthetic gene clusters of these phytotoxins have been reported, such as ascochitine and solanapyrone (45, 46). Genomic analysis of putative SM biosynthetic gene clusters (SMGCs) indicated that *Ascochyta* spp. harbor relatively fewer SMGCs than other plant fungal pathogens (47, 48). Amino acid sequence comparisons of backbone proteins for each SMGC revealed that more than one-half of the SMGCs are shared in this genus. Moreover, each species also has its own characteristics. For instance, the SMGC of solanapyrone exists only in *A. rabiei*, but the ascochitine biosynthetic gene cluster was absent only in this species. Importantly, our results showed that all 26 identified SMGCs in *A. pisi* may be involved in fungal virulence based on the transcriptome *in planta*. Identifying the secondary metabolism and constructing mutants in SMGCs of *A. pisi* will be used to further analyze the pathogenicity of this fungus.

## MATERIALS AND METHODS

### Fungal isolation, identification, and pathogenicity test.

Strain HNA23 was isolated from an infected pea leaf with typical symptoms of ascochyta blight in China (30.25N, 120.21E) according to a protocol described previously (3). Subsequently, HNA23 was purified using a single spore and cultured on complete medium (CM) (1% glucose, 0.2% peptone, 0.1% yeast extract, 0.1% Casamino Acids, nitrate salts, trace elements, 0.01% vitamins, 20 g of agar, and 1 L of water [pH 6.5]) for morphological identification. The production of pycnidia was studied on 1/2 CM plates. After 10 days of incubation at 25°C, the conidia were stained with calcofluor white (a fluorescent dye that binds to cell walls) (catalog no. 18909; Sigma-Aldrich, St. Louis, MO) and visualized using the Leica (Wetzlar, Hesse-Darmstadt, Germany) TCS SP5 imaging system. For molecular identification, ITS, RPB2, TUB2, and LSU were amplified and sequenced using previously reported primer pairs (10). A phylogenetic analysis was performed using MEGA7 software with 1,000 bootstrap replicates.

For the pathogenicity test, the pea leaves, stems, and pods were sterilized and inoculated with mycelial discs (0.5 cm in diameter) of HNA23. Inoculated samples were incubated in a growth chamber at 25°C with 100% humidity, with a 12-h photoperiod. The disease symptoms of inoculated samples were imaged at 3 days postinoculation. Each treatment comprised five biological replicates.

### Genome sequencing, *de novo* assembly, and annotation.

High-quality genomic DNA was extracted from HNA23 and sequenced. Genome sequencing was performed by Novogene Co., Ltd., using the 150-bp paired-end Illumina HiSeq and single-molecule, real-time (SMRT) PacBio sequencing platforms. The *A. pisi* HNA23 genome assembly was produced from PacBio SMRT reads using CANU assembler (v2.2) (49) and corrected and refined using Illumina short-read data with NextPolish (50). The mitochondrial genome was assembled based on sequence homology with mitochondrial DNA from other fungal species. Telomeres were annotated by manual observation of tandem TTAGGG repeat sequences at the ends of contigs in the final assembly (34). Sequencing statistics of the corrected PacBio assembly for HNA23 were generated using QUAST v4.6.2 (51). Genome annotation was performed using BRAKER2 pipelinev (v2.1.5) (52). RNA-seq data were filtered using Trimmomatic v0.39 (53) to remove low-quality sequences and subsequently mapped to the HNA23 genome using STAR aligner (v2.7.6) (54) to facilitate genome annotation. To evaluate the quality of the genome assembly and annotation, BUSCO v5.2.2 (55) was used with the lineage data set dothideomycetes_odb10 (56). BLAST v2.2.29 (57) was used to align the sequences of predicted genes/gene products against the nonredundant (Nr) database. GO analysis was performed using Blast2GO v2.5 (58) based on Nr hits. OrthoFinder v2.5.2 (59) was then used to determine the conserved families in all 7 species and the unique families in individual species based on gene family colinear relationships.

### Molecular phylogenetic analysis.

The available genomes of *Ascochyta* spp. were retrieved from the NCBI database and evaluated using BUSCO v5.2.2 for phylogenetic analysis, including *A. fabae* (NCBI accession no. GCA_004335285.1), *A. koolunga* (accession no. GCA_004151165.1), *A. lentis* (accession no. GCA_004011705.1), *A. rabiei* (accession no. GCF_004011695.1), *A. viciae-villosae* (accession no. GCA_004335205.1), and *A. viciae* (accession no. GCA_004335155.1). The single-copy orthologs (3,783 genes) conserved across all 7 *Ascochyta* species were retrieved and subjected to multiple-nucleotide-sequence alignment using MAFFT v7.471 (60) and trimAl v1.4 (61). After alignment, a maximum likelihood phylogenetic tree based on the concatenated sequences of all single-copy orthologs was constructed using IQ-TREE v1.6.12 (62) with 1,000 bootstrap replicates.

### CAZymes and secretome prediction.

CAZymes were identified using dbCAN2 (63). Proteins of each species were analyzed using SignalP v5.0 (64) and TMHMM v2.0 (65) for the prediction of the secretory signal peptide and the transmembrane domain. Proteins containing a signal peptide but without a transmembrane domain were selected as classical secretory proteins. Protein sequences lacking the signal peptide and transmembrane domain were further analyzed using SecretomeP v2.0 (66) to screen for nonclassical secretory proteins. The predicted secretome was functionally annotated by assigning GO terms using ShinyGO v0.75.

For the identification of small secreted cysteine-rich effectors, all secreted proteins were further subjected to EffectorP 2.0 to identify putative effectors. Next, small secreted cysteine-rich effectors were obtained based on the criteria that the effector consists of ≤200 aa and contains ≥2% cysteine residues.

### Prediction and analysis of secondary metabolite gene clusters.

Secondary metabolite gene clusters (SMGCs) were predicted using the Web-based antiSMASH fungal server v6.0 (67). A comparison of the best hits for *A. pisi* SMGCs in the other 6 species was conducted based on the average percent identity of amino acid sequences of the backbone proteins as previously described (68). SMGC backbone proteins in *A. pisi* were compared with all SMGC backbone proteins in other species by BLASTP. Subsequently, an average backbone protein identity was calculated per cluster; the best hit is shown in a heat map. Backbone proteins are defined as proteins with the annotations PKS(-like), NRPS(-like), hybrid, and TC.

### Transcriptome sequencing and analyses.

The mycelial samples during different infection processes on pea leaves were prepared using the sandwiched sample preparation method as previously described (69). Briefly, the pea cultivar Zhongwan 4, which is susceptible to *A. pisi*, was grown in a greenhouse at 25°C. At the seedling stage, the pea leaves were detached and treated with a 0.05% (vol/vol) solution of Tween 20 to improve wetting. The mycelia of HNA23 were harvested after 24 h of incubation with sterilized filter papers and washed with sterile distilled water 3 times. Next, the washed mycelia were laid onto and sandwiched between the upper surfaces of two leaves at 25°C. At 2 h (contact stage) and 8 h (penetration stage) postinoculation, the mycelial mat was carefully peeled from the leaves and preserved in liquid nitrogen. For the lesion formation stage at 20 h postinoculation (hpi), the mycelial mat and visible mycelia in the tissue of pea leaves were collected with sterilized tweezers under a dissecting microscope. Each treatment comprised three biological replicates.

RNA of the samples was extracted using TRIzol reagent (catalog no. 15596026; Invitrogen, Carlsbad, CA) and subjected to RNA-seq using an Illumina HiSeq2000 platform (2× 150-bp paired ends). Raw reads were processed using Trimmomatic v0.39 (53) to remove the low-quality reads and adapter sequences. Next, the generated clean reads were mapped to the genome of HNA23 using STAR v2.7.6a (54). The number of reads mapped to each gene was counted using featureCounts v1.6.0 (70) to perform differential expression analysis. The criterion for a differentially expressed gene was a log_2_ fold change of greater than 1 or less than −1 with an adjusted *P* value of ≤0.05. GO enrichment analysis of differentially under- or overexpressed genes was conducted using ShinyGO v0.75 (71). All statistical analyses were performed in R v.3.6.3.

### Data availability.

The genome assembly for HNA23 was deposited in the National Genomics Data Center (NGDC) database (accession no. GWHBOVI00000000). The RNA-seq data set was deposited in the NCBI Sequence Read Archive database (BioProject accession no. PRJNA887927). All other relevant data are within the manuscript and its supplemental material.
