# Supplementary material for: Genome and Transcriptome Analysis of Ascochyta pisi Provides Insights into the Pathogenesis of Ascochyta Blight of Pea
Source: Microbiol Spectr. 2023 Jan 16;11(1):e04488-22. doi: 10.1128/spectrum.04488-22 (PMC9927284; doi:10.1128/spectrum.04488-22)
Supplement: Supplemental file 1 — Supplemental material. Download spectrum.04488-22-s0001.pdf, PDF file, 0.3 MB [file spectrum.04488-22-s0001.pdf]

SUPPLEMENTAL MATERIAL

Figure S1. Phylogenetic tree constructed using Maximum likelihood method. Four house-keeping genes (ITS, RPB2, TUB2 and LSU) were used.

Figure S2. GO functional analysis of *A. pisi* HNA23.

Figure S3. KEGG functional analysis of *A. pisi* HNA23.

Figure S4. KOG functional analysis of *A. pisi* HNA23.

Figure S5. Gene expression profiles in *A. pisi* during the infection on pea leaves. (A) Heatmap of normalized FPKM of all expressed genes in HNA23. The Z-score represents the deviation from the mean by standard deviation units. Red and green colors indicate up-regulated and down-regulated genes during interaction, respectively. FPKM: fragments per kilobase of transcript per million fragments mapped. (B-C) Volcano plot of DEGs in the comparison between contact stage (2-hour post-inoculation, hpi) and penetration stage (8 hpi) (B), penetration stage (8 hpi) and lesion formation stage (20 hpi) (C). X-axis indicates $\log_2$ (fold change) of DEGs between two compared stages. Y-axis indicates the $-\log_{10}$ (*P* adjusted value of gene expression variations). The up-regulated, down-regulated, and no-different genes are dotted in red, blue, and grey, respectively. (D) Veen diagram of up-regulated genes in groups CS 2 hpi vs PS 8 hpi and CS 2 hpi vs LFS 20 hpi.

Figure S6. *A. pisi* unique gene expression profiles during the infection on pea leaves. (A) Heatmap of normalized FPKM of all expressed unique genes in A. pisi HNA23. The Z-score represents the deviation from the mean by standard deviation units. Red and green colors indicate up-regulated and down-regulated genes during interaction, respectively. FPKM: fragments per kilobase of transcript per million fragments mapped. (B) mRNA expression patterns of up-regulated unique genes during infection process.

Figure S7. Comparative expression levels of *A. pisi*-specific SSCPs and conserved SSCPs during infection process. Log_2(FPKM) value of each gene encoding SSCP at 2 hpi, 8 hpi and 20 hpi were indicated. The significance between *A. pisi*-specific SSCPs and conserved SSCPs was statistically analyzed using the Wilcoxon rank-sum test ($P \leq 0.05$). ns: no significant difference.

Supplementary file 1. Gene family analysis of 7 *Ascochyta* species

Supplementary file 2. Summary of CAZymes in HNA23 and other *Ascochyta* species

Supplementary file 3. Information of RNA-seq data in this study

Supplementary file 4. Differentially expressed genes in *A. pisi* HNA23 during the infection on pea leaves

Supplementary file 5. Unique gene expression patterns during contact stage and lesion formation stage (2 hpi vs 20 hpi)

Supplementary file 6. Expression profiles of 245 CAZymes in *A. pisi* HNA23 during the infection on pea leaves

Supplementary file 7. List of putative secreted proteins in 7 *Ascochyta* species

Supplementary file 8. Expression profiles of secreted proteins in *A. pisi* HNA23 during the infection on pea leaves

Supplementary file 9. Information of 74 putative SSCPs in *A. pisi* HNA23

Supplementary file 10. Biosynthetic gene clusters of secondary metabolites in *A. pisi* HNA23

Figure S1

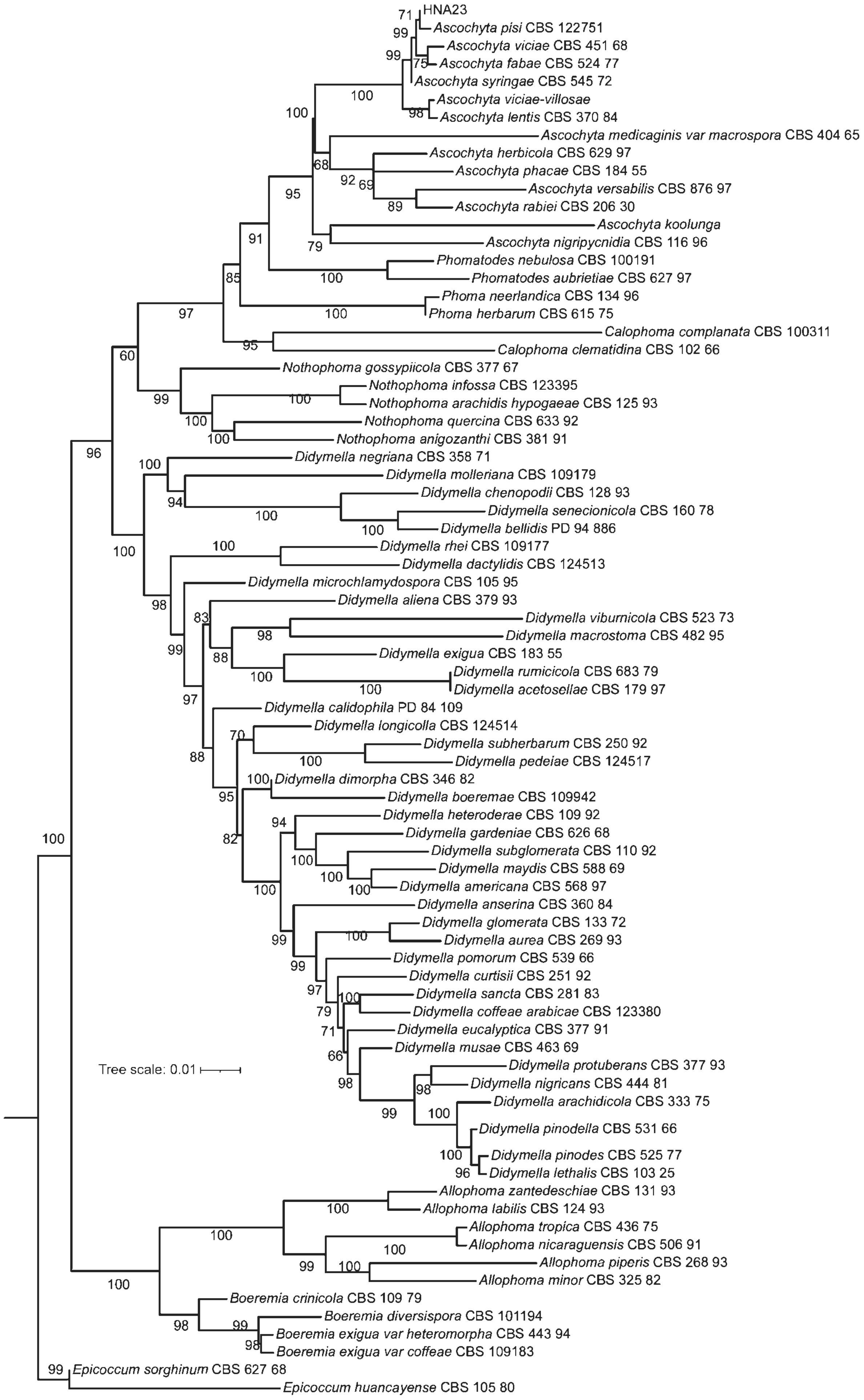

HNA23
Ascochyta pisi CBS 122751
Ascochyta viciae CBS 451 68
Ascochyta fabae CBS 524 77
Ascochyta syringae CBS 545 72
Ascochyta viciae-villosae
Ascochyta lentis CBS 370 84
Ascochyta medicaginis var macrospora CBS 404 65
Ascochyta herbicola CBS 629 97
Ascochyta phacae CBS 184 55
Ascochyta versabilis CBS 876 97
Ascochyta rabiei CBS 206 30
Ascochyta koolunga
Ascochyta nigripycnidia CBS 116 96
Phomatodes nebulosa CBS 100191
Phomatodes aubrietiae CBS 627 97
Phoma neerlandica CBS 134 96
Phoma herbarum CBS 615 75
Calophoma complanata CBS 100311
Calophoma clematidina CBS 102 66
Nothophoma gossypiicola CBS 377 67
Nothophoma infossa CBS 123395
Nothophoma arachidis hypogaeae CBS 125 93
Nothophoma quercina CBS 633 92
Nothophoma anigozanthi CBS 381 91
Didymella negriana CBS 358 71
Didymella molleriana CBS 109179
Didymella chenopodii CBS 128 93
Didymella senecionicola CBS 160 78
Didymella bellidis PD 94 886
Didymella rhei CBS 109177
Didymella dactylidis CBS 124513
Didymella microchlamydospora CBS 105 95
Didymella aliena CBS 379 93
Didymella viburnicola CBS 523 73
Didymella macrostoma CBS 482 95
Didymella exigua CBS 183 55
Didymella rumicicola CBS 683 79
Didymella acetosellae CBS 179 97
Didymella calidophila PD 84 109
Didymella longicolla CBS 124514
Didymella subherbarum CBS 250 92
Didymella pedeiae CBS 124517
Didymella dimorpha CBS 346 82
Didymella boeremae CBS 109942
Didymella heteroderae CBS 109 92
Didymella gardeniae CBS 626 68
Didymella subglomerata CBS 110 92
Didymella maydis CBS 588 69
Didymella americana CBS 568 97
Didymella anserina CBS 360 84
Didymella glomerata CBS 133 72
Didymella aurea CBS 269 93
Didymella pomorum CBS 539 66
Didymella curtisii CBS 251 92
Didymella sancta CBS 281 83
Didymella coffeae arabicae CBS 123380
Didymella eucalyptica CBS 377 91
Didymella musae CBS 463 69
Didymella protuberans CBS 377 93
Didymella nigricans CBS 444 81
Didymella arachidicola CBS 333 75
Didymella pinodella CBS 531 66
Didymella pinodes CBS 525 77
Didymella lethalis CBS 103 25
Allophoma zantedeschiae CBS 131 93
Allophoma labilis CBS 124 93
Allophoma tropica CBS 436 75
Allophoma nicaraguensis CBS 506 91
Allophoma piperis CBS 268 93
Allophoma minor CBS 325 82
Boeremia crinicola CBS 109 79
Boeremia diversispora CBS 101194
Boeremia exigua var heteromorpha CBS 443 94
Boeremia exigua var coffeae CBS 109183
Epicoccum sorghinum CBS 627 68
Epicoccum huancayense CBS 105 80
Tree scale: 0.01

Figure S2

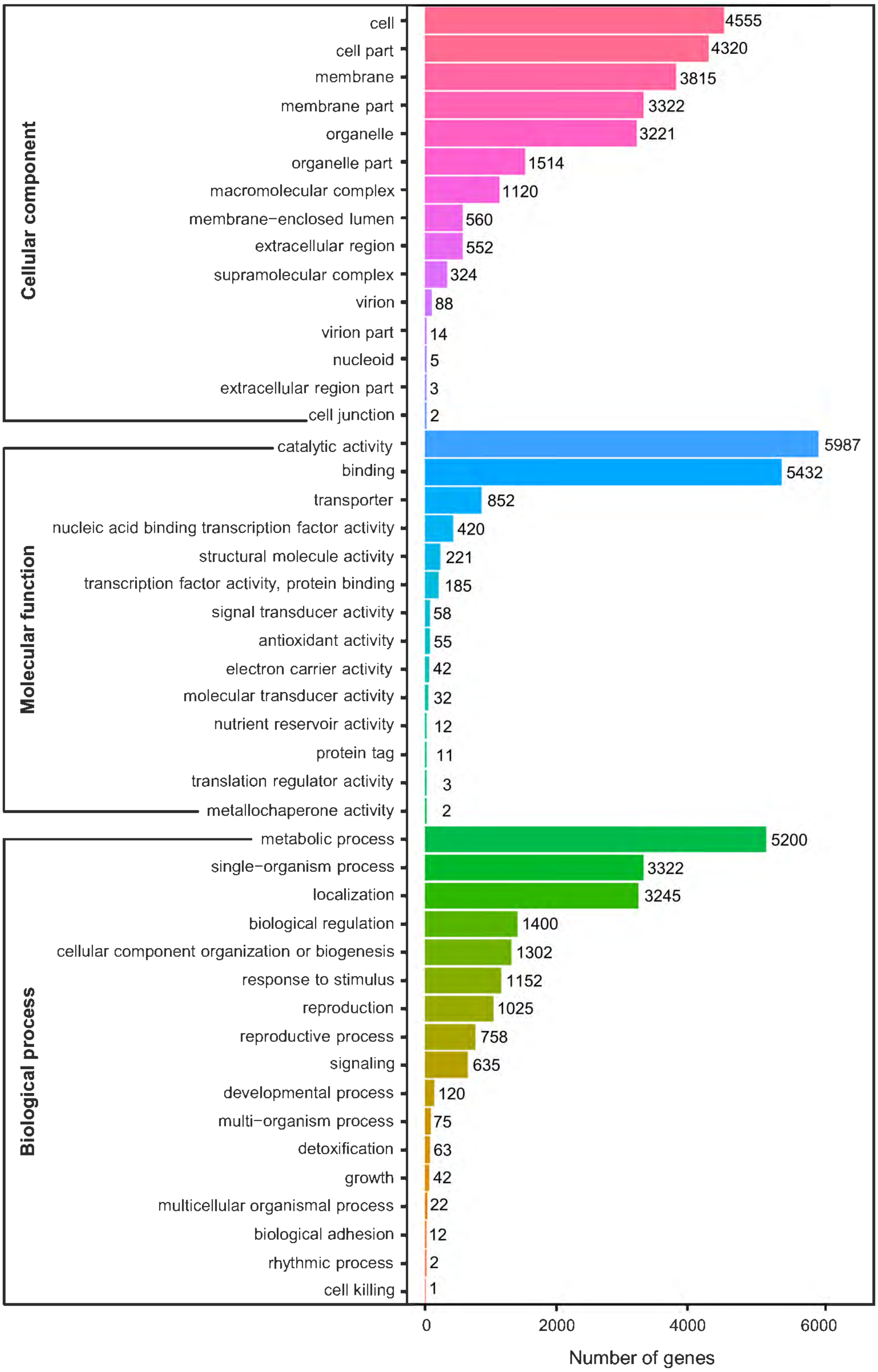

Cellular component
cell 4555
cell part 4320
membrane 3815
membrane part 3322
organelle 3221
organelle part 1514
macromolecular complex 1120
membrane−enclosed lumen 560
extracellular region 552
supramolecular complex 324
virion 88
virion part 14
nucleoid 5
extracellular region part 3
cell junction 2
Molecular function
catalytic activity 5987
binding 5432
transporter 852
nucleic acid binding transcription factor activity 420
structural molecule activity 221
transcription factor activity, protein binding 185
signal transducer activity 58
antioxidant activity 55
electron carrier activity 42
molecular transducer activity 32
nutrient reservoir activity 12
protein tag 11
translation regulator activity 3
metallochaperone activity 2
Biological process
metabolic process 5200
single−organism process 3322
localization 3245
biological regulation 1400
cellular component organization or biogenesis 1302
response to stimulus 1152
reproduction 1025
reproductive process 758
signaling 635
developmental process 120
multi−organism process 75
detoxification 63
growth 42
multicellular organismal process 22
biological adhesion 12
rhythmic process 2
cell killing 1
0
2000
4000
6000
Number of genes

Figure S3

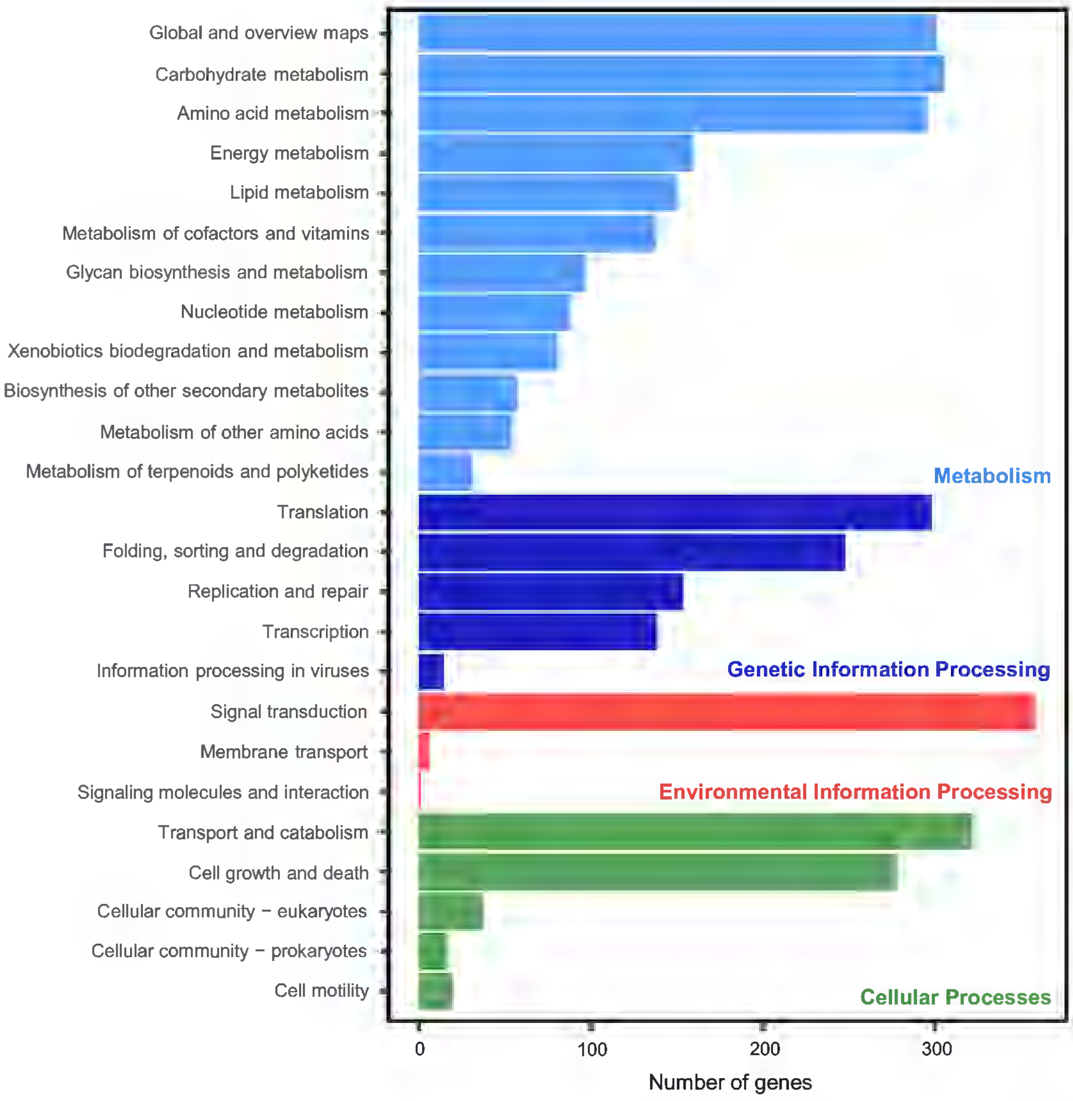
Global and overview maps
Carbohydrate metabolism
Amino acid metabolism
Energy metabolism
Lipid metabolism
Metabolism of cofactors and vitamins
Glycan biosynthesis and metabolism
Nucleotide metabolism
Xenobiotics biodegradation and metabolism
Biosynthesis of other secondary metabolites
Metabolism of other amino acids
Metabolism of terpenoids and polyketides
Translation
Folding, sorting and degradation
Replication and repair
Transcription
Information processing in viruses
Signal transduction
Membrane transport
Signaling molecules and interaction
Transport and catabolism
Cell growth and death
Cellular community − eukaryotes
Cellular community − prokaryotes
Cell motility
Metabolism
Genetic Information Processing
Environmental Information Processing
Cellular Processes
0
100
200
300
Number of genes

Figure S4

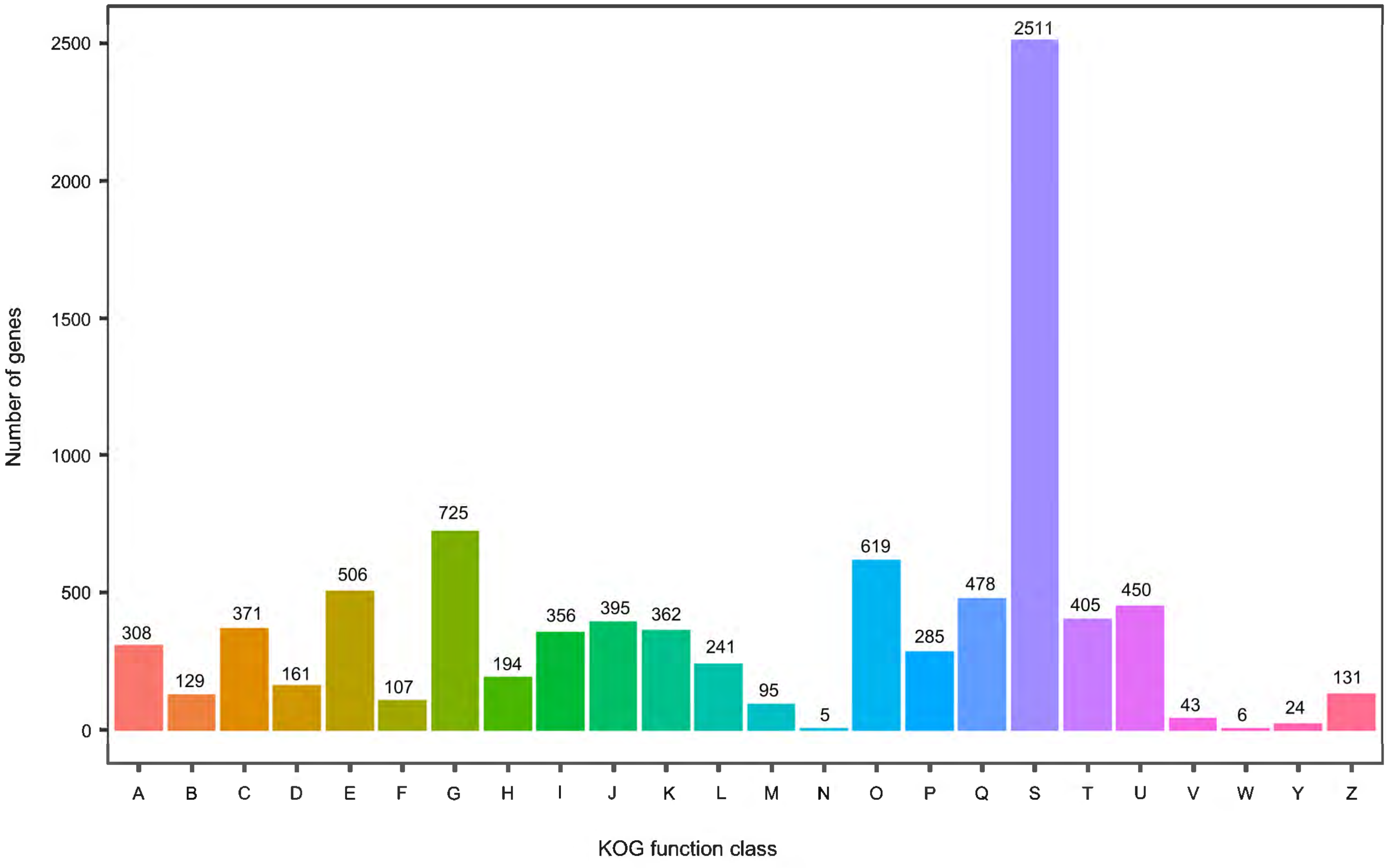

[A] RNA processing and modification
[B] Chromatin structure and dynamics
[C] Energy production and conversion
[D] Cell cycle control, cell division, chromosome partitioning
[E] Amino acid transport and metabolism
[F] Nucleotide transport and metabolism
[G] Carbohydrate transport and metabolism
[H] Coenzyme transport and metabolism
[I] Lipid transport and metabolism
[J] Translation, ribosomal structure and biogenesis
[K] Transcription
[L] Replication, recombination and repair
[M] Cell wall/membrane/envelope biogenesis
[N] Cell motility
[O] Posttranslational modification, protein turnover, chaperones
[P] Inorganic ion transport and metabolism
[Q] Secondary metabolites biosynthesis, transport and catabolism
[S] Function unknown
[T] Signal transduction mechanisms
[U] Intracellular trafficking, secretion, and vesicular transport
[V] Defense mechanisms
[W] Extracellular structures
[Y] Nuclear structure
[Z] Cytoskeleton

Figure S5

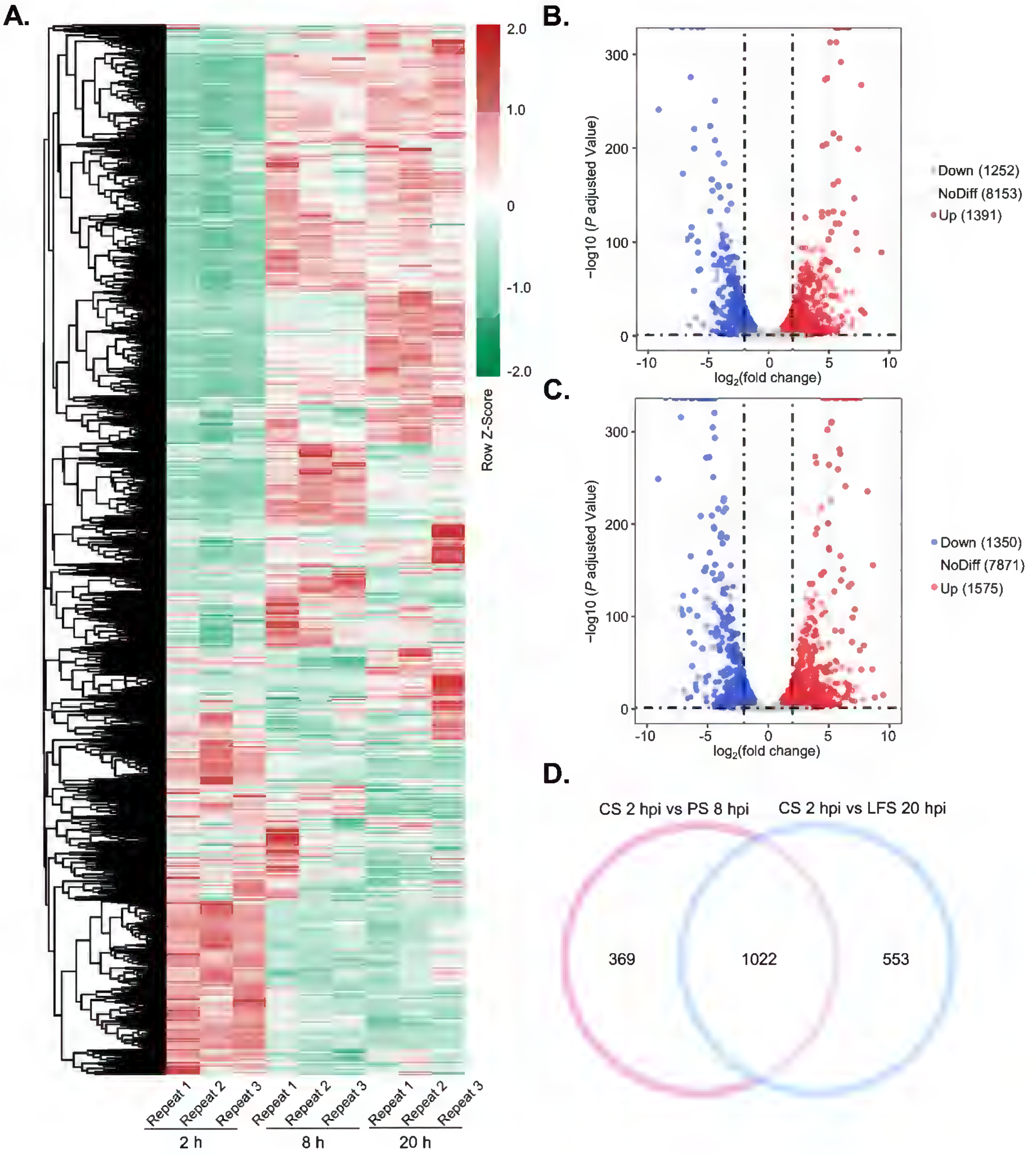
A.
2.0
1.0
0
-1.0
-2.0
Row Z-Score
Repeat 1
Repeat 2
Repeat 3
Repeat 1
Repeat 2
Repeat 3
Repeat 1
Repeat 2
Repeat 3
2 h
8 h
20 h
B.
300
200
100
0
-10
-5
0
5
10
log2(fold change)
−log10 (P adjusted Value)
Down (1252)
NoDiff (8153)
Up (1391)
C.
300
200
100
0
-10
-5
0
5
10
log2(fold change)
−log10 (P adjusted Value)
Down (1350)
NoDiff (7871)
Up (1575)
D.
CS 2 hpi vs PS 8 hpi
CS 2 hpi vs LFS 20 hpi
369
1022
553

Figure S6

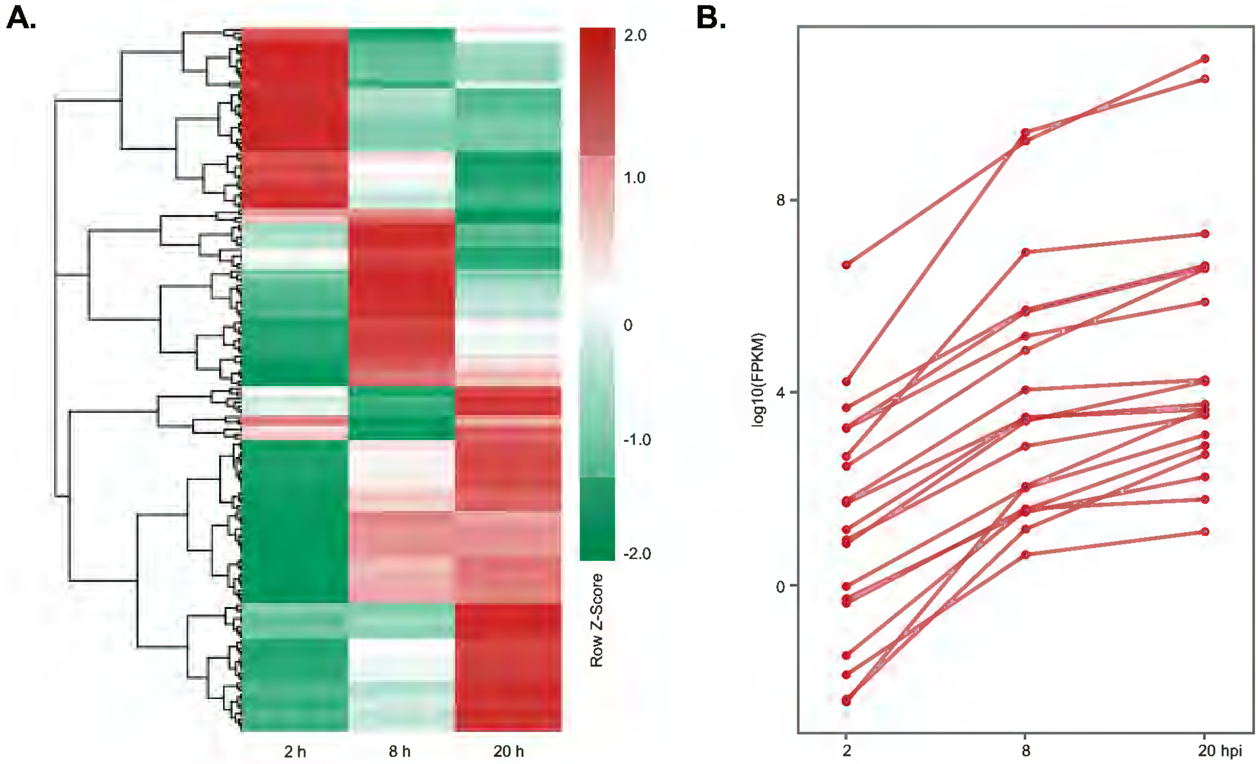

Figure S6 *A. pisi* unique gene expression profiles during the infection on pea leaves. (A) Heatmap of normalized FPKM of all expressed unique genes in *A. pisi* HNA23. The Z-score represents the deviation from the mean by standard deviation units. Red and green colors indicate up-regulated and down-regulated genes during interaction, respectively. FPKM: fragments per kilobase of transcript per million fragments mapped. (B) mRNA expression patterns of significant up-regulated unique genes during infection process.

Figure S7

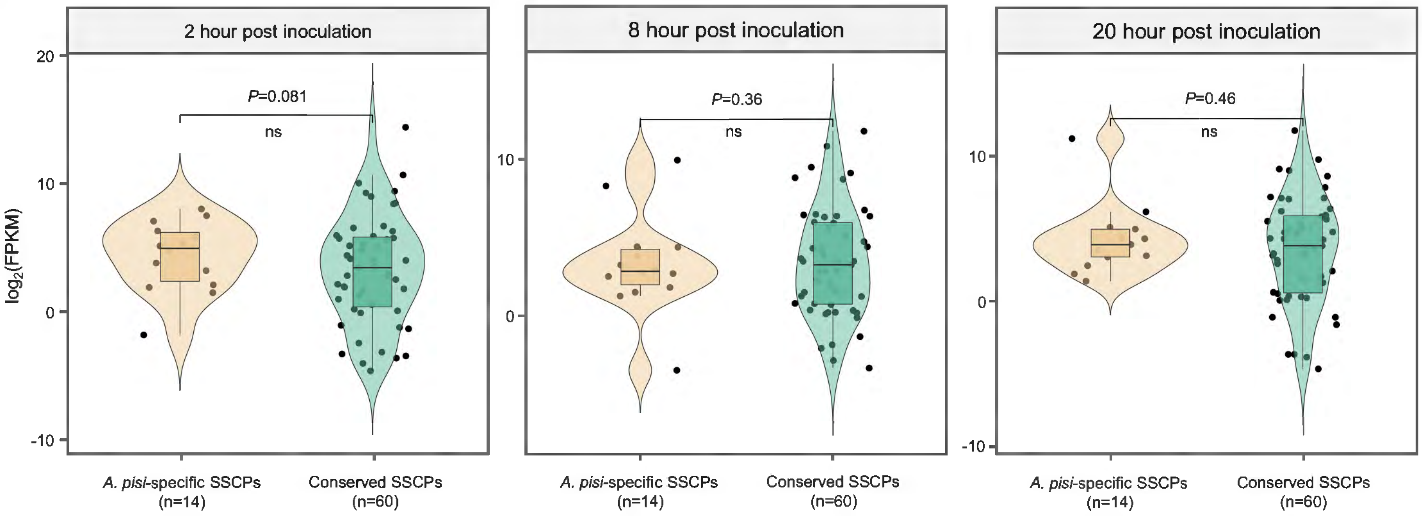

2 hour post inoculation
8 hour post inoculation
20 hour post inoculation
log2(FPKM)
P=0.081
P=0.36
P=0.46
ns
20
10
0
-10
A. pisi-specific SSCPs (n=14)
Conserved SSCPs (n=60)

Supplementary file 1. Gene family analysis of 7 *Ascochyta* species

Organism Name	Strain	NCBI accession	Gene family no.	Clustered gene no.	Common gene no.	Unique gene no.	
						Clustered	Nonclustered
A. pisi	HNA23	PRJNA887707	10,053	10,791	7,659	22	201
A. fabae	247/15	GCA_004335285.1	11,843	12,688	7,678	4	273
A. koolunga	PIN-15-415	GCA_004151165.1	9,601	10,568	7,750	11	1,459
A. lentis	Al4	GCA_004011705.1	10,718	11,488	7,606	207	150
A. rabiei	Me14	GCF_004011695.1	9,763	10,747	7,558	43	510
A. viciae-villosae	ONG-16-641	GCA_004335205.1	11,509	12,743	7,758	71	657
A. viciae	FOR-16-616	GCA_004335155.1	11,913	12,790	7,698	2	400

Supplementary file 2. Summary of CAZymes in HNA23 and other *Ascochyta* species

Specie	AAs	CBMs	CEs	GHs	GTs	PLs	Total
A. pisi HNA23	106	5	75	258	77	34	555
A. viciae	109	6	71	256	75	33	550
A. fabae	105	8	69	251	75	35	543
A. lentis	118	4	83	271	85	34	595
A. viciae-villosae	111	8	70	258	79	36	562
A. rabiei	102	3	78	244	79	32	538
A. koolunga	93	9	59	239	68	30	498

Supplementary file 3. Information of RNA-seq data in this study

Infection stage	Biological replicate	Total raw reads	Total high quality clean reads	Mapping to genome of HNA23		
				Genome mapped reads	Gene mapped reads	Expressed genes
Contact stage (2 hpi)	1	10,657,175	10,403,739	10,355,845 (99.53%)	8,965,528 (86.2%)	10,414
	2	14,142,993	14,104,551	14,041,175 (99.55%)	12,117,277 (85.9%)	10,446
	3	16,036,879	16,016,162	15,934,810 (99.49%)	14,107,376 (88.1%)	10,451
Penetration stage (8 hpi)	1	14,824,902	14,814,460	14,712,573 (99.31%)	12,107,653 (81.7%)	10,559
	2	14,849,276	14,786,634	14,647,483 (99.06%)	12,072,952 (81.6%)	10,490
	3	14,878,070	14,862,813	14,776,100 (99.42%)	12,202,558 (82.1%)	10,483
Lesion formation stage (20 hpi)	1	15,828,362	15,749,309	15,625,885 (99.22%)	12,827,904 (81.5%)	10,482
	2	17,762,405	17,300,317	17,158,816 (99.18%)	14,202,682 (82.1%)	10,517
	3	17,781,064	17,716,196	17,677,700 (99.78%)	14,439,298 (81.5%)	10,539
